# Twelve Children with Varicella Vaccine Meningitis: Neuropathogenesis of Reactivated Live Attenuated Varicella Vaccine Virus

**DOI:** 10.3390/v12101078

**Published:** 2020-09-25

**Authors:** Ethan H. Heusel, Charles Grose

**Affiliations:** Virology Laboratory, Children’s Hospital, University of Iowa, Iowa City, IA 52242, USA; ethan-heusel@uiowa.edu

**Keywords:** varicella-zoster virus, herpes zoster, herpes vaccine, VZV gC, viral meningitis, latency, trigeminal ganglion, RNA polymerase III

## Abstract

Varicella vaccine is a live attenuated varicella-zoster virus (VZV). Like its parental strain called VZV pOka, the vaccine virus vOka retains some neurotropic properties. To better understand vOka neuropathogenesis, we reassessed 12 published cases of vOka meningitis that occurred in once-immunized and twice-immunized children, all of whom had bouts of herpes zoster preceding the central nervous system infection. Eight of the 12 meningitis cases occurred in children who had received only one immunization. There was no pattern to the time interval between varicella vaccination and the onset of herpes zoster with meningitis. Four of the meningitis cases occurred in children who had received two immunizations. Since all four children were 14 years old when meningitis was diagnosed, there was a strong pattern to the interval between the first vaccination at age 1 year and onset of meningitis, namely, 13 years. Knowledge of pathogenesis requires knowledge of the location of herpes zoster; the majority of dermatomal rashes occurred at sites of primary immunization on the arm or thigh, while herpes zoster ophthalmicus was uncommon. Based on this literature review, currently there is no consensus as to the cause of varicella vaccine meningitis in twice-immunized children.

## 1. Introduction

Four cases of varicella vaccine meningitis in twice immunized adolescents have been reported in the United States since 2017 [1]. The live attenuated varicella vaccine (strain vOka) has been administered to children in the United States for 35 years [2,3]. Universal varicella vaccination is now recommended in several other countries [4]. The effectiveness of the varicella vaccination program is excellent, and the disease varicella (chickenpox) caused by the wild type varicella has virtually disappeared [5,6]. The safety profile of varicella vaccine is also excellent [7]. The most serious complication in healthy children is probably central nervous system infection, which is caused by reactivation of the vaccine virus from the dorsal root ganglia (DRG), usually years after the first varicella vaccination. 

The goal of this review is to present an overview of varicella vaccine meningitis in children immunized with the live attenuated varicella vaccine. This review includes a re-assessment of conclusions that we published in 2012 [8]. In 2012, there were seven known cases of varicella vaccine meningitis, all of which had occurred in children who had received only one varicella vaccination. The varicella vaccine was approved by the U.S.A. Food and Drug Administration in 1995 as a single-dose regimen around age 1 year. In 2006, the recommendation for varicella vaccination was changed from one to two doses, usually administered at age 1, and then before entry to primary school (4–6 years) [9]. Subsequently, there was a belief that complications such as varicella vaccine meningitis would not occur in children who had received two varicella vaccinations. Since that assumption has proven not to be true, we have undertaken a second investigation of varicella vaccine meningitis, in order to include children who have received either one or two varicella vaccinations. Reports of the first four cases of varicella vaccine meningitis in twice-immunized adolescents were published in 2017, 2019 and 2020 [1,10,11]. We also review the literature on varicella meningitis caused by reactivation of wild type varicella in children, because these wild-type cases provide insight into the pathogenesis of varicella vaccine meningitis.

## 2. Varicella Vaccine Meningitis after Herpes Zoster in Once-Immunized Children

**Figure 1:**
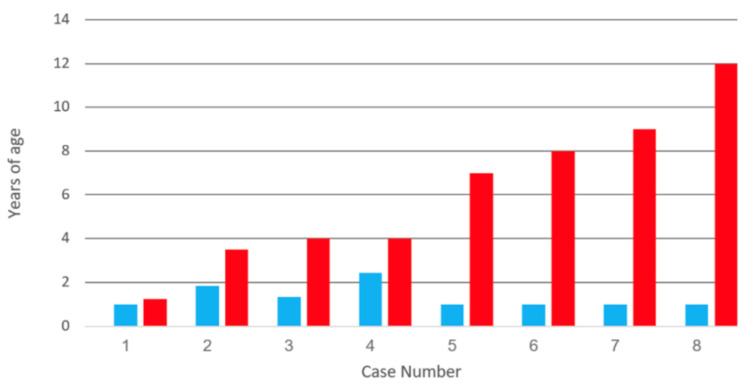
Duration between varicella vaccination and varicella vaccine meningitis in eight children. All children had received one varicella vaccination, after which they later developed herpes zoster and meningitis. Data from references [8,12,13]. Note the absence of pattern to the time intervals between vaccination and meningitis. Age of varicella vaccination: blue bar; age of meningitis: red bar.

In our review published in 2012, we located seven cases in the literature [8]. We have now cited an additional case for a total of eight cases of varicella vaccine meningitis in children who have received one varicella vaccination (Figure 1 and Table 1) [12,13]. The virology studies for most children listed in Table 1 were performed at the Centers for Disease Control, Atlanta, Georgia. When Figure 1 is examined, it is apparent that there is no trend relating to average time for meningitis to occur after the first and only vaccination. Two of the children developed varicella vaccine meningitis shortly after a diagnosis of cancer. Since chemotherapy for cancer is known to cause VZ reactivation, this result is expected. In the other six children, there was no diagnosis of immunosuppression. The shortest time interval between vaccination and varicella vaccine meningitis in an immunocompetent child was 22 months. The longest time interval between varicella vaccination and meningitis in an immunocompetent child was 11 years.

All children had herpes zoster preceding onset of meningitis. However, there is an unusual aspect about the dermatomal distribution, namely six of eight zoster rashes were in a cervical dermatome. The typical distribution for herpes zoster following wild type varicella includes dermatomes T3 though T10 and cranial nerve V (trigeminal ganglion) [14,15]. Child #1 was known to have had his vaccination in the thigh and he developed herpes zoster at his injection site in a lumbar dermatome. The site of immunization for most of the other cases was not reported. When the varicella vaccine was first approved in the United States, there was a long catch-up period when older children could be immunized. It is most likely that several of the children included in Table 1 were immunized in the arm rather than the thigh because they were older; thus, they developed herpes zoster in the cervical dermatomes. The current recommendation is for all children to have their first varicella vaccination in the thigh around age 1 year.

## 3. Varicella Vaccine Meningitis after Herpes Zoster in Twice-Immunized Children

**Figure 2:**
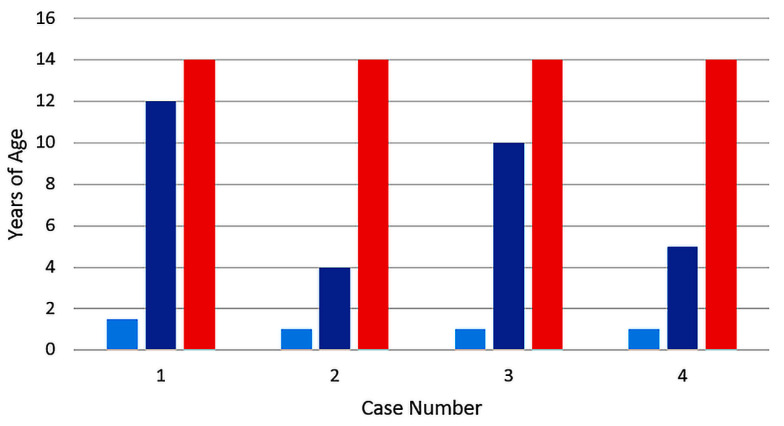
Duration between varicella vaccination and varicella vaccine meningitis in four children. All children had received two varicella vaccinations. All four children developed herpes zoster and meningitis 13 years after their first vaccination. Data from references [1,10,11]. Age of first varicella vaccination: light blue bar; age of second vaccination: dark blue bar; age of meningitis: red bar.

**Figure 3:**
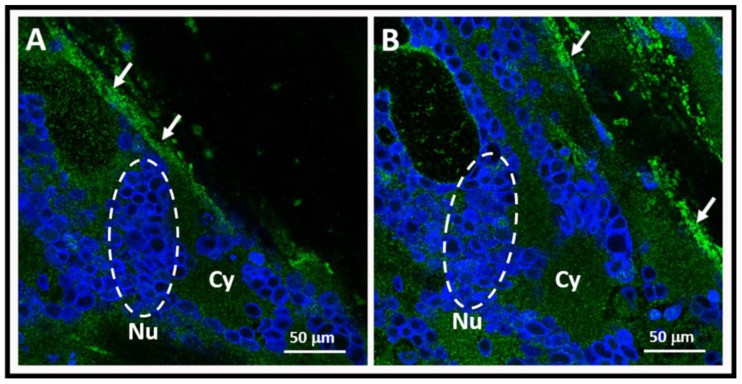
Varicella antibody in the cerebrospinal fluid. Varicella antibody was detected by a fluorescent antibody to membrane antigen test. The test was carried out by confocal microscopy imaging of varicella-zoster virus (VZV) infected monolayers. Two confocal images are shown (**A** and **B**). Clusters of nuclei (Nu; blue circles) within the cytoplasm (Cy) of an infected cell are encircled by a dashed white line. Arrows point to positive fluorescent antibody attaching to VZV antigens in the plasma membrane. The majority of the positivity resides in the plasma membranes of the infected cells because that is where the majority of viral glycoproteins are located. One viral target is VZV glycoprotein gE.

Varicella vaccine meningitis has been reported in four twice-immunized children since 2017; three of the children were immunocompetent and one had cancer (Figure 2 and Table 2). In contrast to children who had received only one immunization, the time interval between the first vaccination and the onset of varicella vaccine meningitis was constant. This interesting point is made apparent in the colored bar graph (Figure 2). Note that all four children were 14 years old; therefore, they were 13 years beyond their first varicella vaccination. All had herpes zoster proceeding onset of meningitis. Since the two-dose regimen for varicella vaccination began in 2006 in the United States, and since the interval between first dose and meningitis was 13 years, these cases are among the initial cohort of children that received two varicella vaccinations. Case #4 had a more detailed virology evaluation at the University of Iowa, which included the detection of the VZV antibody in the CSF (Figure 3). Detection of VZV antibody in the CSF is a sensitive assay by which to diagnose varicella meningitis [16,17]. At least one VZV laboratory has reported that detection of the VZV antibody in CSF may be a more sensitive assay for VZV meningoencephalitis than the detection of VZV DNA in CSF [18].

## 4. Pathogenesis of Varicella Vaccine Meningitis

**Figure 4:**
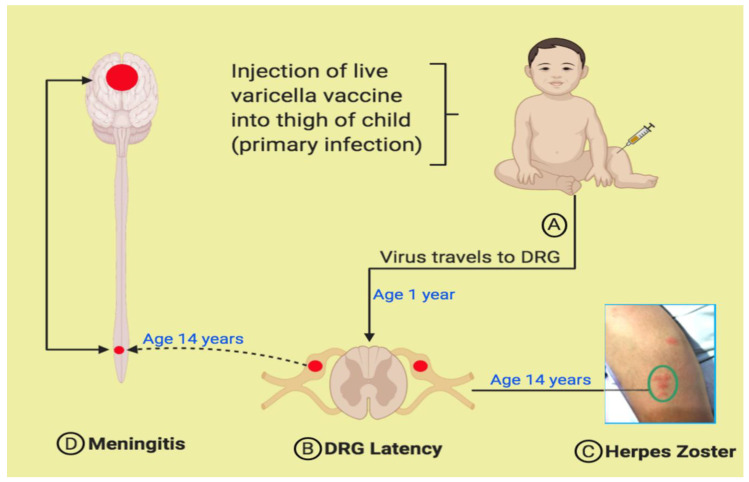
Pathogenesis of varicella meningitis in an immunized child. After a varicella vaccination, the virus is transported via sensory nerves from the skin (**A**) to the lumbar dorsal root ganglion (DRG) (**B**). When the virus reactivates from the DRG, the virus is carried to the lumbar dermatome in the thigh (**C**). The rash in Panel C is the herpes zoster rash of case #4 in Table 2. The virus is also carried into the spinal cord, where meningitis ensues (**D**).

To date, the cases described in Table 1 and Table 2 gave a history of herpes zoster proceeding or concomitant with onset of symptoms of acute meningitis. After review of prior models [19], we propose a pathogenesis model (Figure 4). Every child around the age of 1 year is given an initial injection of the live attenuated varicella vaccine in the thigh (Figure 4A). Virus replicates locally in the skin; some viral particles enter the free endings of A and C nociceptor fibers, after which the viral particles are transported to the soma of lumbar DRG, where the virus enters a latent state (Figure 4B). (A second vaccine injection can be given before the child enters primary school). Several years later, the vaccine virus reactivates from the DRG, after which the virus is carried in the same pseudounipolar fibers anterograde to the thigh (distal process), presenting as herpes zoster in a lumbar dermatome on the leg (Figure 4C; vesicles are encircled). In turn, the virus can also travel proximally from the DRG to the gray matter of the spinal cord, where replication occurs (Figure 4D). From the lumbar spinal cord, the virus can be transported to the brain via interneurons within the spinothalamic tract.

The cerebrospinal fluid indices are shown in Table 2. When compared with cerebrospinal fluid studies performed decades ago, in which adults with herpes zoster were evaluated with a lumbar puncture, the indices were similar as, for example, between 38–46% had a pleocytosis [20,21]. Although PCR analyses were not performed in the earlier studies, pleocytosis in the CSF probably indicated detectable VZV in the CSF. Thus, the live attenuated varicella vaccine virus has retained the neurotropism of its wild-type counterpart.

Because the vaccine virus is attenuated, the question has arisen whether reactivation of the vaccine virus can lead to severe or prolonged herpes zoster. The impression from the literature is that herpes zoster caused by the vaccine virus is invariably mild, but that is not always the case [22]. Two reports have described severe cases of cutaneous herpes zoster caused by varicella vaccine virus [23,24]. Since we have seen severe cases, we propose the following explanation that is based on a pseudorabies animal model of neuronal infection. Pseudorabies virus is the herpes virus of swine; one neuronal infection called “mad itch” is dermatomal and, therefore, is a good animal model for herpes zoster [25,26].

The vaccine virus replicates in the skin after reactivation. Thereafter, progeny viruses enter sensory neurons and are transported retrograde to the DRG within 1–2 days. In turn, these viruses replicate in the DRG and are transported anterograde to the skin near the site of the original herpes zoster rash within 1–2 days. Other vaccine viruses are transported from the DRG to the spinal column. Altogether, therefore, within one episode of VZV reactivation, before a strong adaptive immune response, there are two or more round trips of the virus transported between DRG and skin or between DRG and spinal cord.

## 5. Varicella Vaccine Meningitis after Herpes Zoster Ophthalmicus

**Figure 5:**
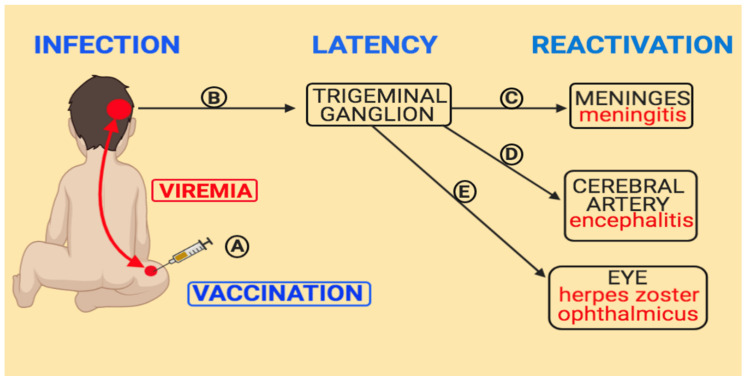
Pathogenesis of varicella vaccine meningitis following herpes zoster ophthalmicus. Following varicella vaccination of about 50% of young children, virus is carried within lymphocytes throughout the body (**A**). Some virus enters and become latent in the trigeminal ganglion (**B**). When this virus reactivates, it can be carried via afferent fibers to the meninges (**C**), cerebral arteries (**D**) and eye (herpes zoster ophthalmicus (**E**).

Varicella vaccine meningitis after herpes zoster ophthalmicus (HZO) deserves a special mention. The trigeminal ganglion can be considered the DRG equivalent for sensory relays to the head and face. As with the DRG, pseudounipolar sensory neurons transmit stimuli from nociceptors in the face and head, including the meninges, to the soma in the trigeminal ganglion [27]. Meningitis after HZO occurred in one case cited in Table 1 [28]. Another case of severe HZO caused by the varicella vaccine virus has been published, but a lumbar puncture was never performed [24]. Since the first varicella vaccination is administered in the thigh, the details within Figure 4 do not apply to VZV latency within the trigeminal ganglion and subsequent HZO. Instead, we have prepared another pathogenesis figure (Figure 5). Around 50% of children have a viremia after their first varicella vaccination [29]. The vaccine virus is carried in the blood stream throughout the body and can infect DRG far beyond the lumbar area and establish distant sites of latency; by this means, the virus passes through the arterial blood supply to the trigeminal ganglion, especially the ophthalmic branch [30]. When the virus reactivates within the ophthalmic branch of the trigeminal ganglion (HZO), the virus can be transported anterogradely along afferent fibers to the meninges (dura mater, arachnoid mater and pia mater), the cerebral arteries or the eye [31].

When the eight cases in Table 1 and the four cases in Table 2 are reviewed, it is apparent that the dermatomal distribution of herpes zoster caused by varicella vaccine virus is different than herpes zoster caused by wild-type virus, as has been previously observed in the United States [32]. One of the most thorough dermatomal mapping studies ever carried out by a single physician in the same general medical practice over a 16-year period (1947–1962), before varicella vaccination, recorded that around 15% of herpes zoster is HZO in both children and adults [14]. Out of the 12 published cases of herpes zoster meningitis caused by the vaccine virus, only one case was HZO. The most likely explanation is apparent in Figure 5. In almost all children with wild-type varicella, the exanthem begins at the hairline and forehead [33]. Thereafter, the virus is directly carried from these early vesicles via the sensory nerves to the ophthalmic branch of the trigeminal ganglion. Over 80% of trigeminal ganglia contain latent VZV in people who have experienced the disease varicella [34]. Since there is no facial exanthem after varicella vaccination and since a viremia occurs in just 50% of vaccinees, far fewer vaccinated children would be susceptible to infection of the trigeminal ganglion and subsequent HZO.

## 6. Varicella Meningitis Caused by Wild Type Varicella

**Figure 6:**
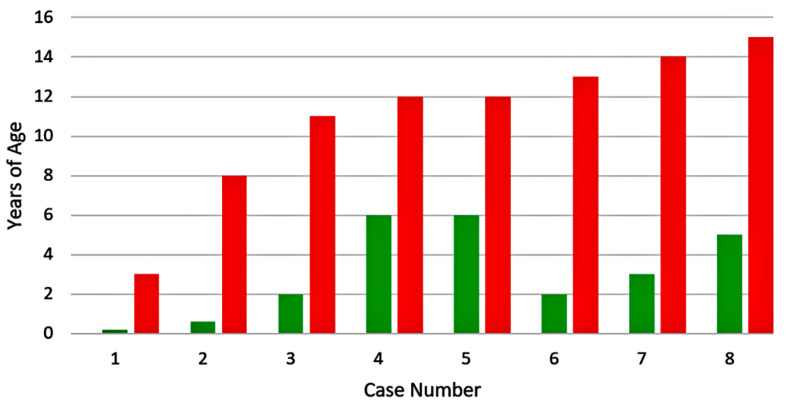
Eight cases of varicella meningitis caused by wild type virus. Each child had herpes zoster prior to developing meningitis. Data from references [36,37]. Age of wild type varicella: green bar; age of meningitis: red bar.

The focus of this review is the varicella vaccine virus. However, for comparison, we include a short summary of varicella meningitis caused by wild type virus. We will mainly discuss cases in immunocompetent children because VZV easily reactivates during several chemotherapy regimens for cancer [15]. First of all, varicella meningitis as a symptomatic complication of herpes zoster in immunocompetent children is very rare. In a 20-year retrospective analysis of herpes zoster cases in children seen at the Mayo Clinic before approval of the varicella vaccine, no case of meningitis was recorded in the medical records [35]. A similar study was performed among the pediatric oncology patients at the University of Iowa Hospital before the approval of the varicella vaccine [15]. Even in children with cancer, no cases of varicella meningitis were observed following herpes zoster even though herpes zoster commonly occurred in this cohort. In spite of the earlier observations from these two medical centers, a small number of cases have since been published about immunocompetent children with herpes zoster who developed meningitis [36,37]. Among eight cases, there were six between the ages of 11–15 years, all of whom had wild type varicella at age 2 years or later (Figure 6). There were two cases with herpes zoster and meningitis at 3 years and 8 years of age; these children had wild type varicella at 2 months and 6 months of age, respectively.

**Figure 7:**
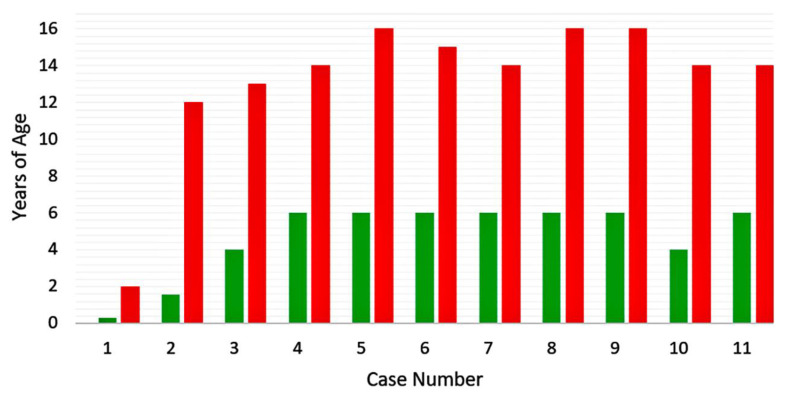
Eleven cases of varicella meningitis caused by wild type virus. None of the children had herpes zoster prior to developing meningitis. Data from reference [38]. Age of wild type varicella: green bar; age of meningitis: red bar.

A recent review of aseptic meningitis in children in Ireland reported 11 cases of VZV meningitis or meningoencephalitis in immune competent children with a prior history of childhood varicella but no herpes zoster [38]; in other words, these 11 children presumably had VZV reactivation in the CNS without clinical herpes zoster (Figure 7). Of interest, 10 of the 11 children were adolescents between ages 12–16. One child was just 2 years old, but that child had a primary varicella infection under age 1. This subgroup from Figure 6 and Figure 7 is known to have earlier VZV reactivation, probably because a suboptimal and short-lived VZV-specific immune response occurs in babies exposed to varicella in utero or in the first months of life [39,40].

Undoubtedly, there are other cases of varicella meningitis in the medical literature. Clinical studies of VZV infections in the central nervous system that included both children and adults sometimes did not differentiate the study population by age and, therefore, were not further assessed in this review [41].

## 7. Comparison between Wild Type vs. Vaccine-Type Varicella Meningitis

It is known from epidemiology studies that herpes zoster occurs less commonly in children who are immunized as compared with children who have wild type varicella [22,32]. It is known from basic virology studies on cell culture and severe combined immunodeficient mouse models that the varicella vaccine virus establishes latency similarly to wild type varicella virus, but the vaccine virus has a lower rate of reactivation [42,43]. Although the varicella vaccine virus reactivates less frequently than the wild type virus, when varicella vaccine virus enters the spinal cord and replicates in the meninges, the initial symptoms and signs of viral meningitis appears to be similar between vaccine-related and wild type-related CNS disease. All cases of varicella vaccine meningitis shown in Figure 1 and Figure 2 were treated with intravenous acyclovir.

The only difference that we have noted in this review is that there are a sizable number of cases of varicella meningitis caused by wild type virus in adolescents who have never experienced clinical herpes zoster, while none have been described to date with the vaccine virus. Presumably, the source of the virus for wild type meningitis in the absence of a herpes zoster rash is the trigeminal ganglion, a common site of latency after wild-type varicella [14]. We have also reported a similar case of an immunocompetent 23-year old who developed wild type VZV meningoencephalitis confined to the temporal lobe in the absence of clinical herpes zoster [44]. He had had wild type varicella at age 6 months. We presume that he had subclinical HZO without rash as the source of his VZV brain infection. Perhaps an explanation is implied in Figure 5 because very few immunized children have latent virus in the trigeminal ganglion, and meningitis following reactivation of vaccine virus in the trigeminal ganglion without a rash is statistically unlikely to occur.

## 8. Conclusions

**Figure 8:**
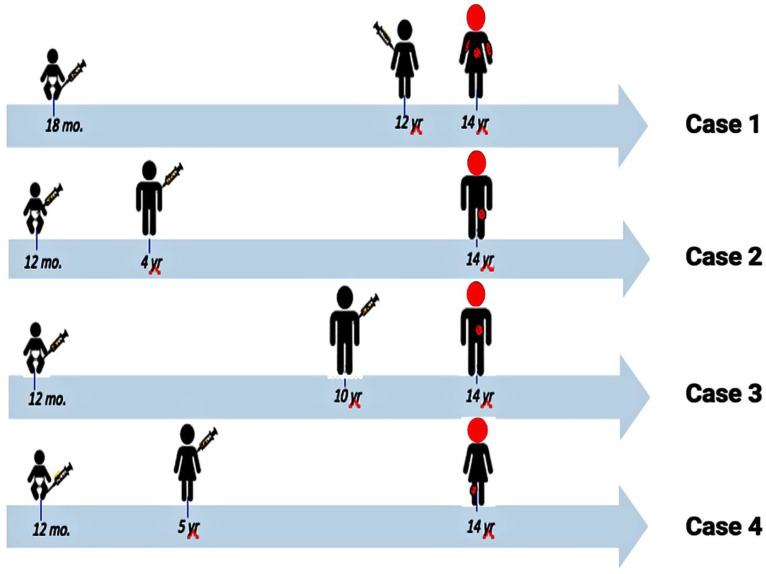
Similarities among the four twice-immunized adolescents who developed varicella vaccine meningitis. All four were given their first varicella vaccination between 12–18 months of age. All four manifested herpes zoster with meningitis at the age of 14 years (heads colored red). Thus, there was a pattern between time of first varicella vaccination and onset of meningitis (~13 years). Data from reference [1].

Staff scientists at the Merck Company published a comprehensive 22-year review of post-marketing safety data of the varicella vaccine in 2019 [45]. The authors noted that more than 200 million doses of the vaccine have been administered worldwide since 1995. Their review confirms the overall safety profile for the vaccine. We concur with all their conclusions made in 2019, when they had recorded 10 cases where the vaccine virus was detected in the cerebrospinal fluid of immunocompetent children with disseminated disease. As noted earlier in this review, we are now aware of a total of 12 children who developed varicella vaccine meningitis as a complication of vaccine-related herpes zoster; the 12 include four twice immunized adolescents (Figure 8).

**Figure 9:**
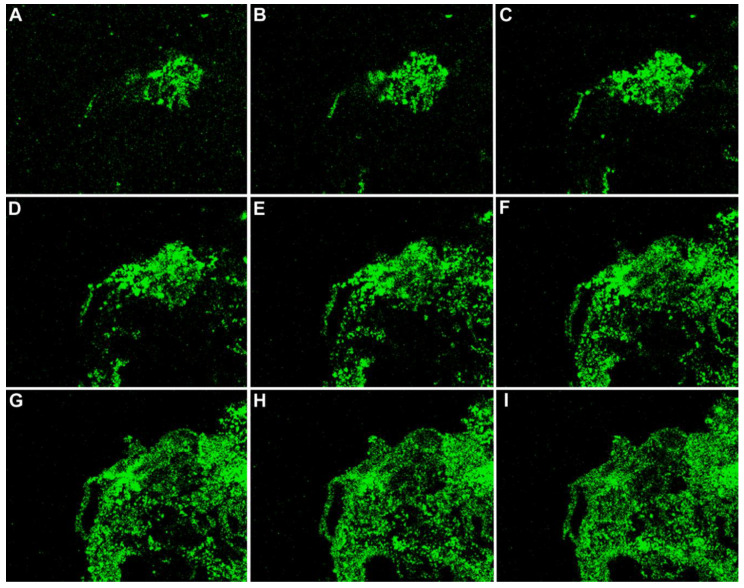
Vesicle of wild type herpes zoster. The VZV glycoprotein gC is a component in the envelope of a VZ virion; every vesicle is filled with virions. The protein is immunolabeled green by a monoclonal antibody probe. The nine micrographs show images of the abundant VZV gC protein from serial sections cut through a herpes zoster vesicle from an edge to the center (**A**–**I**). Magnification = 200×.

At the current time, there is no consensus as to the explanation for cases of varicella vaccine meningitis in immunized and immunocompetent children. We have considered these possible explanations. (i) Waning immunity. We point to a 7-year seroepidemiology study of 10,000 female and male Air Force recruits (2008–2015), in which the military personnel who had received varicella vaccination were 24% less likely to be VZV seropositive than recruits who had wild type varicella as children [46]. (ii) Differential immune response to varicella vaccination vs. the disease varicella. In addition to possible quantitative differences in duration of antibody responses to varicella vaccination vs. the disease varicella, there are also qualitative differences. In particular, there is a lessened antibody response to the major VZV structural glycoprotein gC in immunized children [47,48]. The VZV gC protein is a major component of wild-type VZV virions found abundantly in skin vesicles and, therefore, the protein is easily visualized by laser scanning confocal microscopy (Figure 9). During infection after vaccination, there is little or no formation of vesicles in the skin; therefore, much less VZC gC is produced [49,50,51]. (iii) Emergence of wild type alleles in the viral genome during reactivation of varicella vaccine virus as herpes zoster. In 2019, we published a case report of severe herpes zoster cases caused by the varicella vaccine virus. When we sequenced portions of the viral genome, we observed that the open reading frame (ORF) 0 had reverted to a wild type allele [23]. Therefore, herpes zoster was more severe in this child. Based on this finding, we hypothesize that the T560C mutation in the VZV ORF0 stop codon did not occur by chance; instead, this mutation (*130R) is likely to be a major determinant of attenuation [52,53]. (iv) The fourth option is a counterbalance to option 3. Rather than changes in the viral genome that enhance vaccine virulence, we hypothesize that there may be a very small number of humans with mutated alleles that predispose them to a more severe VZV infection by diminishing a VZV-centered innate immune response. The Casanova laboratory first provided proof-of-principle that single-gene inborn errors of immunity can lead to herpes simplex virus 1 encephalitis in children. They showed that herpes encephalitis may occur in otherwise healthy children with a previously undetected deficiency of Toll-like receptor 3 (TLR-3) [54]. In turn, TLR-3 deficiency led to decreased interferon alpha/beta and interferon gamma-mediated immunity in the brain [55]. Subsequently the Mogensen laboratory provided a similar proof-of-principle that a single-gene inborn error of RNA polymerase III can lead to severe VZV meningoencephalitis in children and adults [56]. RNA polymerase III is a sensor of foreign DNA in the cytosol (for example, VZV DNA); in that role, the RNA polymerase has been described as a gatekeeper to prevent serious VZV infections [57]. Because detection of these alleles in the human genome usually requires whole exome sequencing, the prevalence of the inborn errors of immunity in children among the general population is not yet known [58]. We encourage whole exome sequencing studies on future cases of varicella vaccine meningitis in immunocompetent children.

## Figures and Tables

**Table 1:** Vaccine virus meningitis in once-immunized children.

Category	1	2	3	4	5	6	7	8
Vaccination (y)	1	1.8	1.3	2.4	1	1	1	1
Meningitis (y)	1.3	3	4	4	7	8	9	12
Zoster	Lum	Tri	Cer	Cer	Cer	Cer	Cer	Cer
Cancer	Yes	No	No	Yes	No	No	No	No
IV Acyclovir	Yes	Yes	Yes	Yes	Yes	Yes	Yes	Yes
Year	2003	2009	2008	2008	2011	2008	2008	2011
VZV Lab	CDC	UK	CDC	CDC	CDC	CDC	CDC	CDC
Gender	Boy	Girl	NG	NG	Boy	NG	NG	Girl

Abbreviations: Lum = lumbar; Tri = trigeminal; Cer = cervical dermatome; CDC = Centers for Disease Control, USA; UK = University College London; NG = gender not given.

**Table 2:** Prominent features among twice-immunized adolescents with varicella vaccine meningitis.

Category	Case 1 [10]	Case 2 [11]	Case 3 [11]	Case 4 [1]
Geography	Boston	Seattle	Seattle	Des Moines
Gender	Girl	Boy	Boy	Girl
Age 1st vaccine (y)	1.5	~1	~1	~1
Age 2nd vaccine (y)	12	4	10	5
Age of meningitis (y)	14	14	14	14
Location of zoster	T5	L1/L2	T8	L4
Years after 1st vaccine	13	13	13	13
Years after 2nd vaccine	2	10	4	9
CSF Screening PCR	Yes	Yes	Yes	Yes
CSF cell count	568	140	81	775
CSF VZV antibody	Not done	No	Not done	Yes
Acyclovir IV (days)	7	7	27	7
Valacyclovir (days)	14	0	Indefinite	14
Immunocompromised	No	No	Yes	No
Year	2017	2019	2019	2020
VZV Lab	CDC	CDC	CDC	Iowa
